# CDYL for infrared and visible light image dense small object detection

**DOI:** 10.1038/s41598-024-54146-1

**Published:** 2024-02-12

**Authors:** Huixin Wu, Yang Zhu, Shuqi Li

**Affiliations:** 1 North China University of Water Resources and Electric Power, Zhengzhou, 450046 China; 2 Xi’an International Studies University, Xi’an, 710128 China

**Keywords:** Object detection, Infrared and visible light, YOLOv8, Computer vision and FliR_Adas_v2 dataset, Computer science, Information technology

## Abstract

To address the phenomenon of many small and hard-to-detect objects in infrared and visible light images, we propose an object detection algorithm CDYL (Convolution to Fully Connect-ed-Deformable Convolution You only Look once) based on the CFC-DC (Convolution to Fully Connected-Deformable Convolution) module. The core operator of CDYL is CFC-DC, making our model not only have a large effective receptive field in infrared and visible light images, but also have adaptive spatial aggregation conditioned by input and task information. As a result, the CDYL reduces the strict inductive bias of traditional CNNs and has long-range dependence for large kernel convolution as well as adaptive spatial aggregation, deeply mining of edge and correlation information in images to enhance sensitivity to small objects, thereby improving performance in dense small object detection tasks. In order to improve the ability of the CFC-DC module to perceive the detailed information of the image, we use the Mish activation function, which has a wider minima which improves the generalization. The effectiveness as well as the generalization of CDYL is evaluated on an infrared image dataset and an UAV image dataset, and it is compared with other state-of-the-art object detection algorithms. Compared to the baseline network YOLOv8l, our model achieved a 3.0% improvement in mAP0.5 in infrared image detection tasks and a 1.1% improvement in mAP0.5 in visible light image detection tasks. The experimental results show that the proposed algorithm achieves superior average precision values on both infrared and visible light images, while maintaining a light weight. Code is publicly available at https://github.com/yangzhu1/CDYL.

## Introduction

With the application of convolutional neural networks, object detection algorithms have become increasingly mature, achieving significant improvements in both accuracy and speed. However, current well performing conventional algorithms mainly target ideal scenarios under limited conditions, in situations where real environmental factors are constantly changing, the performance is often average, such as the small size of the object in high-altitude or wide-angle view, the complex background, the low quality of the infrared and visible light images due to poor lighting conditions at night, and the presence of blurring in the infrared image when the vehicle is in motion need to be ad-dressed. Moreover, the computation of algorithms is restricted by the limited computing power of edge platforms, which poses challenges for their practical application. There-fore, to ensure its application on edge devices such as drones or cars, it is necessary to improve the generalization ability of object detection algorithms, so that they have ac-curate and stable detection performance in various scenarios.

The object detection algorithms aim to obtain the position and category of targets in the image. Mainstream object detection algorithms use convolutional neural networks and can be divided into two categories: two-stage models based on candidate regions and one-stage models based on regression. The two-stage model generally first generates candidate regions based on the input image, and then classifies and regresses the candidate regions, which usually has higher detection accuracy compared to one-stage model, such as RCNN^1^, Fast R-CNN^2^, Mask R-CNN^3^, etc. However, one-stage model usually transformer the object detection problem into a regression problem, based on global regression classification, so there is no need to generate candidate regions in the stage, and the category and location information of the target can be directly obtained, such as YOLO[^4–9^] series, SSD[^10^] series, RetinaNet^11^, etc. In summary, single-stage algorithms are more suitable for edge devices due to their high detection speed.

The definition of small objects is divided into two categories: absolutely small objects with object pixels less than 32 × 32 in the COCO dataset and relatively small objects with object size less than 10% of the image size. Due to the presence of many dense small targets from the perspective of edge devices such as drones and cars, there are still many problems that need to be solved when applying object detection algorithms in these scenarios.

**Figure 1:**
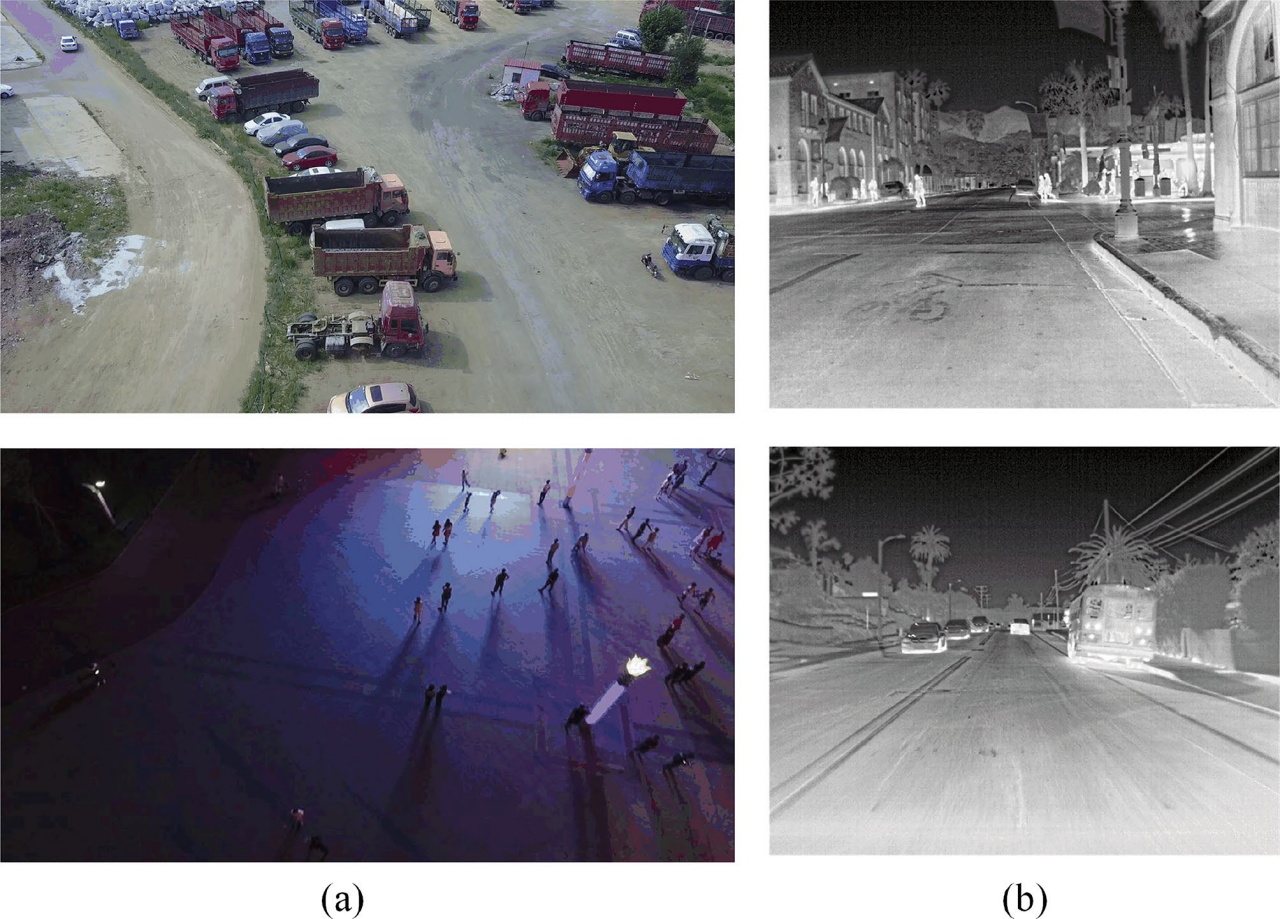
(**a**) Is a visible light image, (**b**) is an infrared image.

Most existing object detection algorithms are developed on visible light image datasets, such as VOC^12^ and COCO^13^, which are greatly affected by lighting. However, there is still a significant gap in the infrared image datasets. When the lighting conditions are good, visible light images have richer texture information than infrared images, but their detection performance is poor when the lighting conditions are not good. As shown in Fig. 1, objects are clear in visible light images during the day, but many objects are invisible in visible light at night, while they are more prominent in infrared images. Due to the fact that infrared imaging devices are not easily affected by light, using infrared images instead of visible light images has become a solution to the problem of low illumination detection. However, compared with visible light images, infrared images have defects such as poor contrast and low resolution. The advantages of the two complement each other, making the synergistic use of visible light and infrared information a more feasible solution. By combining image information from different sensors, the adaptability of object detection algorithms to complex scenes can be improved, thereby improving the accuracy of object detection algorithms.

In recent years, visual transformers (ViTs)^14,15^ have become the preferred choice for large-scale visual basic models. Some pioneers^16–20^ have used ViTs to defeat convolutional neural networks (CNNs) and significantly improve the performance of a wide range of computer vision tasks. Through analysis, it was found that the main reasons why ViTs can defeat CNNS are as follows:

1. From the operator view^21–23^, ViTs is able to learn more powerful and robust representations from massive data than CNNs, because it introduces long-range dependency and adaptive spatial clustering.
2. From the architecture view^21,23,24^, ViTs includes a series of advanced components that are not included in regular CNNs, such as layer normalization (LN)^25^. Although recent work has introduced long-range dependency into CNNs by using dense convolutions with very large kernels(e.g.,31 × 31), there is still a significant gap in performance compared to ViTs.

To address the above issues, in this paper, we investigate the infrared and visible modes of the data, and in this work, we design a new core operator called CFC-DC (Convolution to Fully Connected-Deformable Convolution). Different from recently improved CNNs with very large kernels such as 31 × 31, the core operator of CFC-DC is a deformable convolution^25^ with a common window size of 3 × 3,

1. Its sampling offset is flexible and can dynamically learn appropriate receptive fields (which can be long or short distances) from given data;
2. By adaptively adjusting the sampling offset and modulation scalar based on input data, adaptive spatial aggregation can be achieved, reducing the over induction bias of regular convolution;
3. The convolution window is a common 3 × 3, avoiding the optimization problems and expensive costs caused by large dense kernels. We also improved the activation function of the baseline network by using the Mish^26^ activation function to enhance the network's ability to perceive detailed information.

In summary, our main contributions are as follows:

1. A new core operator CFC-DC is proposed, which can retain the long-range dependencies, realize adaptive spatial aggregation, and improve the performance of object detection.
2. A new activation function is used to improve the network's ability to perceive infrared and visible light image information without increasing the computational cost.
3. We demonstrate that CDYL (Convolution to Fully Connected-Deformable Convolution You only Look once) can efficiently handle both infrared and visible modes of data, enhancing robustness and generalization for small object detection.
4. We propose a new lightweight algorithm, CDYL, which can be applied more efficiently in practice.

## Related work

Traditional object detection algorithms use a combination of sliding windows, feature extractors, and feature classifiers to predict targets. However, due to the limitations of manually designed features, traditional algorithms once stagnated. With the development of large-scale datasets and computing resources, convolutional neural networks have become the mainstream of object detection. On the basis of AlexNet^27^, many deeper and more effective convolutional neural network architectures have been proposed, such as VGG^28^, GoogleNet^29^, ResNet^30^, ResNeXt^31^, Efficient Net^32,33^, etc. In addition to architecture design, more complex convolution operations have also been developed, such as deep convolution^34^ and deformable convolution^35,36^. Deformable convolution has a larger receptive field of view and can retain long-range dependency information, which can improve the problem of losing target features in deeper networks. By referring to the advanced design of transformers, convolutional neural networks have shown good performance in visual tasks, and introducing dynamic weights^37^ with long-range dependency.

In recent years, a new visual foundation model has focused on transformer-based architectures. ViTs is the most representative model, which has achieved great success in object detection tasks due to its global receptive field with long-range dependency. However, the global receptive field is affected by a large amount of computing resources, which limits its application on edge devices. To address this issue, PVT^15,38^ and Linformer^39^ globally focused on the downsampling key and value maps, Deformable convolution is applied in convolutional neural networks, DAT deformably focused on the sparsely sampled information in the value map, HaloNet^40^ and Swin tranformer developed a local attention mechanism to achieve adaptive spatial aggregation. In this work, our goal is to develop a CNN based foundational model that maintains long-range dependencies at low computational costs and achieves adaptive spatial aggregation to better address target detection tasks in infrared and visible light images.

## Methods

**Figure 2:**
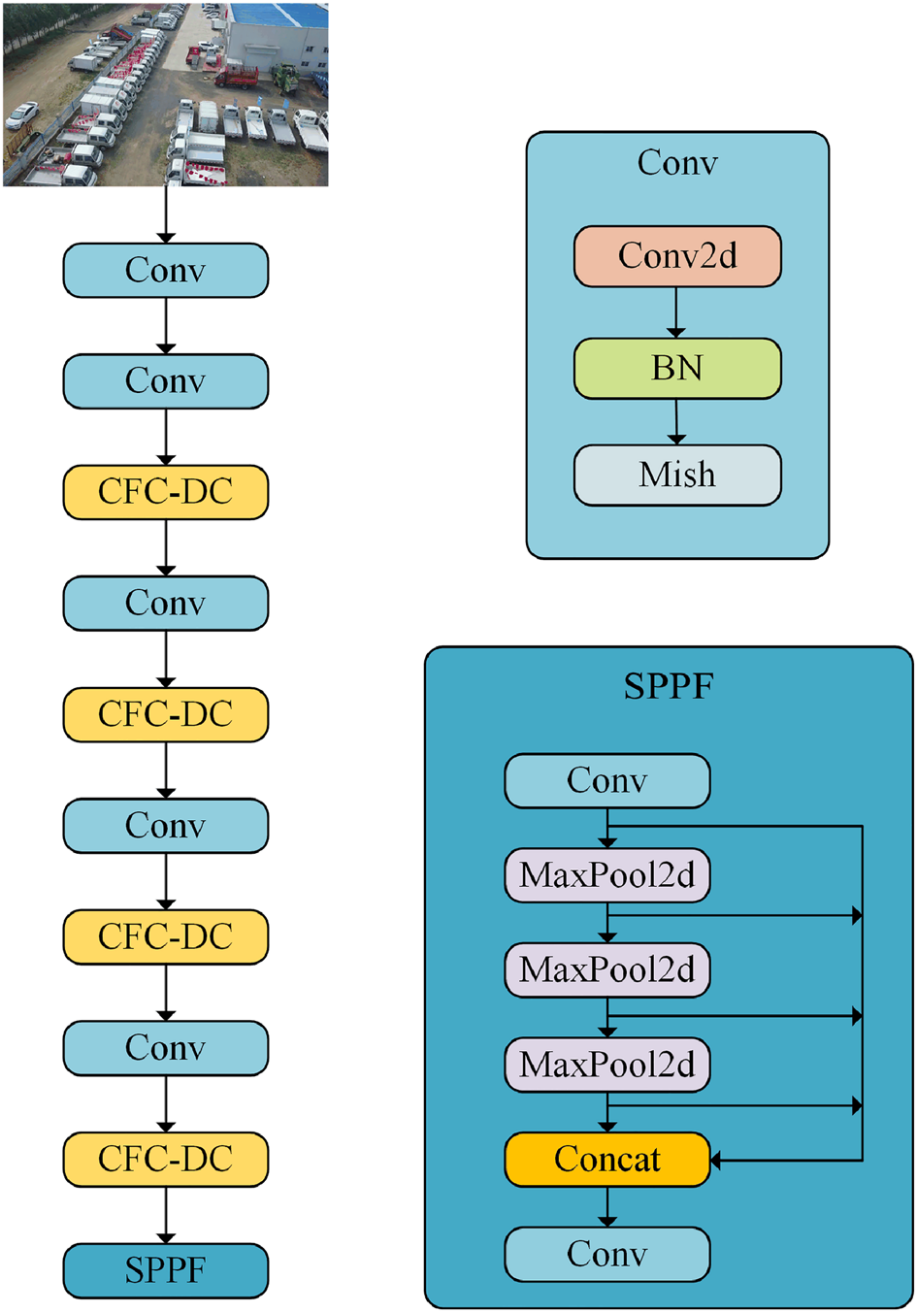
The backbone network structure of CDYL.

In this paper, we propose a new object detection network CDYL(Convolution to Fully Connected-Deformable Convolution You only Look once) with YOLOv8l as the baseline network, and its backbone network is shown in Fig. 2. We use the core operator CFC-DC in the backbone network and neck network, which preserves the long-range dependencies of the image and realizes adaptive spatial aggregation. We also use Mish activation function to adapt the CFC-DC, which improves the ability of CFC-DC to perceive the information, and therefore it can cope with the task of detecting the small objects in the infrared and the visible light images in a better way.

### CFC-DC (convolution to fully connected-deformable convolution)

Usually infrared and visible light images have insufficient small object features, visible light images have low contrast under low illumination, infrared images have low contrast when the ambient temperature is closer to the target, it is difficult to distinguish between the target and the background, and motion blur is produced by moving objects. These factors lead to difficult to detect objects difficult to accurately locate and identify in the image, thus affecting the performance of edge devices in a variety of application scenarios. For increasing the effective receptive field and edge information mining, there are mainly methods such as applying large kernel convolution and increasing network depth. But the large kernel convolution will greatly increase the computational cost.

We all know that models with larger effective receptive field (long-range dependence) usually perform better on downstream visual tasks^41–43^. However, our regular 3 × 3 convolution has a relatively small actual effective receptive field and cannot obtain long-range dependencies, limiting the performance of the network. Not only that, regular convolution has highly-inductive properties and lacks adaptive spatial aggregation, restricting its ability to learn more general and robust patterns from web-scale data.

**Figure 3:**
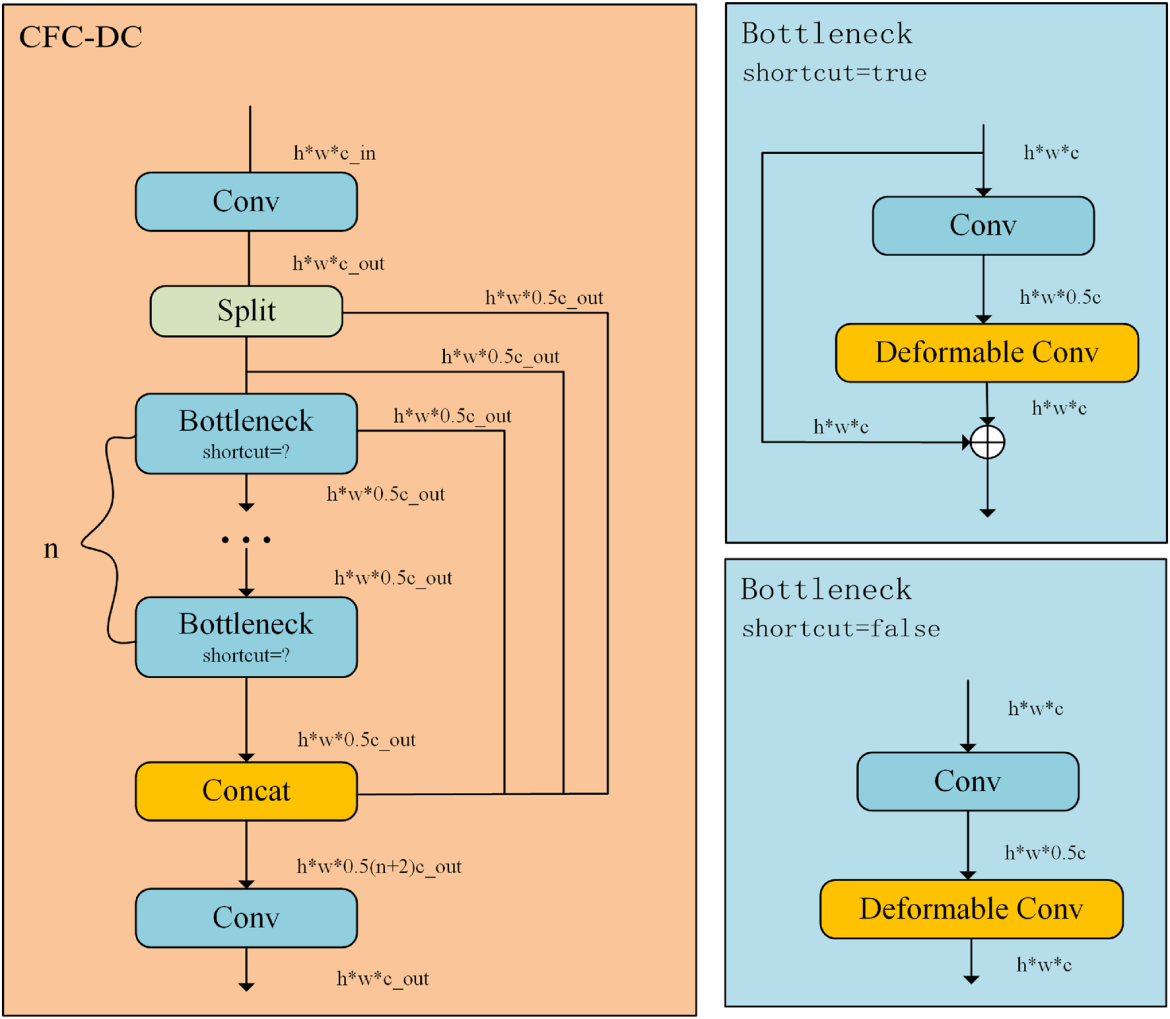
CFC-DC (Convolution to fully connected-deformable convolution) module structure diagram.

To address the above issues, we propose a new core operator CFC-DC. As shown in Fig. 3. We used Deformable Convolution in CFC-DC module, and the sampling offsets and modulation scales are predicted by passing input feature x through a separable convolution(a 3 × 3 depth-wise convolution followed by a linear projection). CFC-DC consists of two convolutional layers and n Bottleneck layers, with two convolutional layers having a kernel size of 1 × 1, a step size of 1, and padding = 0; We used the mish activation function which being unbounded above, and avoiding saturation, which generally causes training to slow down due to near-zero gradients drastically. Mish has smoother output, which means smoother loss phenomena, making it easier for CFC-DC to optimize and better generalize; The bottleneck layer consists of a convolutional layer with a kernel size of 3 × 3 and a step size of 1, and a Deformable Convolution with a kernel size of 3 × 3. The bottleneck layer has an additional parameter shortcut, which is of type bool. When the shortcut is true, the structure is shown in Fig. 3 and applied in the backbone; When the shortcut is FALSE, the structure is shown in Fig. 3 and applied in neck.

CFC-DC solves the shortcomings of regular convolution in terms of long-range dependencies and adaptive spatial aggregation. Inheriting the strict inductive bias of convolution, our model is more efficient with fewer training data and shorter training time. Based on sparse sampling, compared with previous methods such as MHSA and large kernel size of reparameterization, it has higher computational and memory efficiency.

### Deformable convolution

In the task of object detection in infrared and visible light images, we introduced Deformable Convolution. In the deformable convolution:

1. We detach the original convolution weights ${w}_{k}$ into depth-wise and point-wise parts, where the depth-wise part is responsible by the original location-aware modulation scalar ${m}_{k}$, and the point-wise part is the shared projection weights w among sampling points;
2. Introducing multi-group mechanism, we split the spatial aggregation process into G groups, each of which has individual sampling offsets $\Delta {p}_{gk}$ and modulation scale $\Delta {m}_{gk}$, and thus different groups on a single convolution layer can have different spatial aggregation patterns, resulting in stronger features for downstream tasks;
3. To alleviate the instability issues, we change element-wise sigmoid normalization to softmax normalization along sample points. In this way, the sum of the modulation scalars is constrained to 1, which makes the training process of models at different scales more stable.

Deformable convolution can be formulated as Eq. (1):

1 $$y\left( {p_{0} } \right) = \sum\limits_{g = 1}^{G} {\sum\limits_{k = 1}^{K} {w_{g} } } m_{gk} X_{g} \left( {p_{0} + p_{k} + \Delta p_{gk} } \right)$$

where G denotes the total number of aggregation groups. For the g-th group, ${w}_{g}\in {R}^{C\times {C}{\prime}}$ denotes the location-irrelevant projection weights of the group, where ${C}{\prime}=C/G$ represents the group dimension. ${m}_{gk}\in R$ denotes the modulation scalar of the k-th sampling point in the g-th group, normalized by the softmax function along the dimension K. ${X}_{g}\in {R}^{{C}{\prime}\times H\times W}$ represents the sliced input feature map. $\Delta {p}_{gk}$ is the offset corresponding to the grid sampling location ${p}_{k}$ in the g-th group.

### Mish activation function

In the task of object detection in infrared and visible light images, we introduced Deformable Convolution. In order to better adapt to CFC-DC and improve its ability to perceive information, we chose the Mish^26^ activation function, which is a smooth, continuous, self-regularized, and non-monotonic activation function mathematically defined as:

2 $$f(x) = x\tanh ({\text{softplus}} (x)) = x\tanh \left( {\ln \left( {1 + e^{x} } \right)} \right)$$

The value range of Mish is [$\approx -0.31,\infty$], and the first derivative of Mish can be defined as:

3 $$f^{\prime } (x) = \frac{{e^{x} \omega }}{{\delta^{2} }}$$

where $\omega =4\left(x+1\right)+4{e}^{2x}+{e}^{3x}+{e}^{x}(4x+6)$ and $\delta =2{e}^{x}+{e}^{2x}+2$. Mish uses the self-gating property where the non-linear function of the input. Due to the preservation of a small amount of negative information, Mish eliminated by design the preconditions necessary for the Dying ReLU phenomenon. This feature will enable our core operator to have better performance and information flow while preserving long-range dependency, thereby enhancing the network's expressive power.

**Figure 4:**
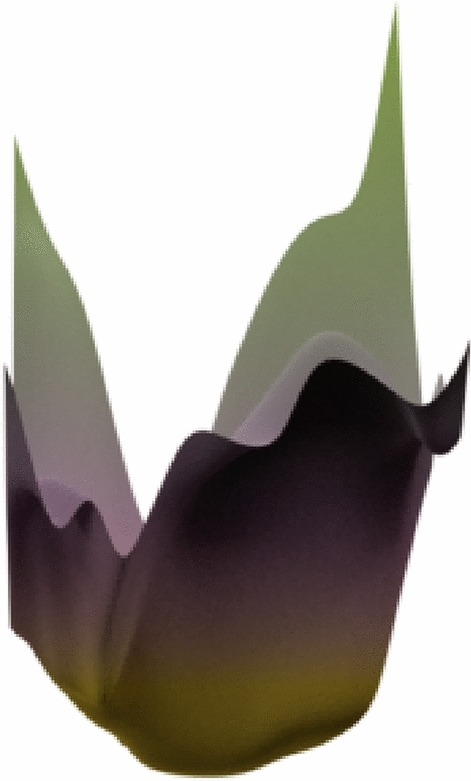
The loss landscapes of Mish.

Having a smooth profile also plays a role in better gradient flow, as shown in Fig. 4, the landscapes were generated by passing in the co-ordinates to a five-layered randomly initialized neural network which outputs the corresponding scalar magnitude. A smooth output landscape means a smooth loss landscape, which will improve the training speed and generalization ability of our network. Therefore, it is more suitable for infrared and visible light scenes.

### VFL (varifocal) loss function

Accurately sorting a large number of candidate detection boxes is crucial for achieving high performance in dense small object detectors. Previous work used classification scores or a combination of classification and prediction localization scores to sort candidate boxes. However, neither of these options will generate reliable rankings, which will reduce detection performance. In this study, we used the VFL loss function^44^, which is expressed as follows:

4 $${\text{VFL}} (p,q) = \left\{ {\begin{array}{*{20}l} { - q(q\log (p) + (1 - q)\log (1 - p))} \hfill & {q > 0} \hfill \\ { - \alpha p^{\gamma } \log (1 - p)} \hfill & {q = 0} \hfill \\ \end{array} } \right.$$

where $p$ is the predicted ICAS(IoU-aware classification score) and $q$ is the object score. For a foreground point, $q$ for its ground-truth class is set as the IoU between the generated bounding box and its gt_IoU and 0 otherwise, whereas for a background point, the object $q$ for all classes is 0.

This loss only reduces the loss contribution from negative examples (*q *= 0) by scaling their losses with a factor of ${p}^{\gamma }$ and does not down-weight positive examples (*q *> 0) in the same way. This is because compared to negative examples, positive examples are extremely rare, and we should retain their valuable learning signals.

## Experiments

In this section, we will provide a detailed introduction to the experiments conducted: In section “datasets” and “Implementation details”, we mainly introduced the dataset and parameter information used in this experiment. In section “Ablation study”, we conducted a series of ablation studies to demonstrate the effectiveness of the proposed algorithm. In section “Comparison of detection results of different object detection algorithms on FliR_Adas_v2 and VisDrone 2019”, We demonstrated the comparison of detection results of different object detection algorithms on FliR_Adas_v2 and VisDrone 2019.

### datasets

We used two datasets to validate the effectiveness and generalization of CDYL for infrared and visible light image detection tasks. We apply LWIR type infrared image detection in the infrared image dataset.

**Figure 5:**
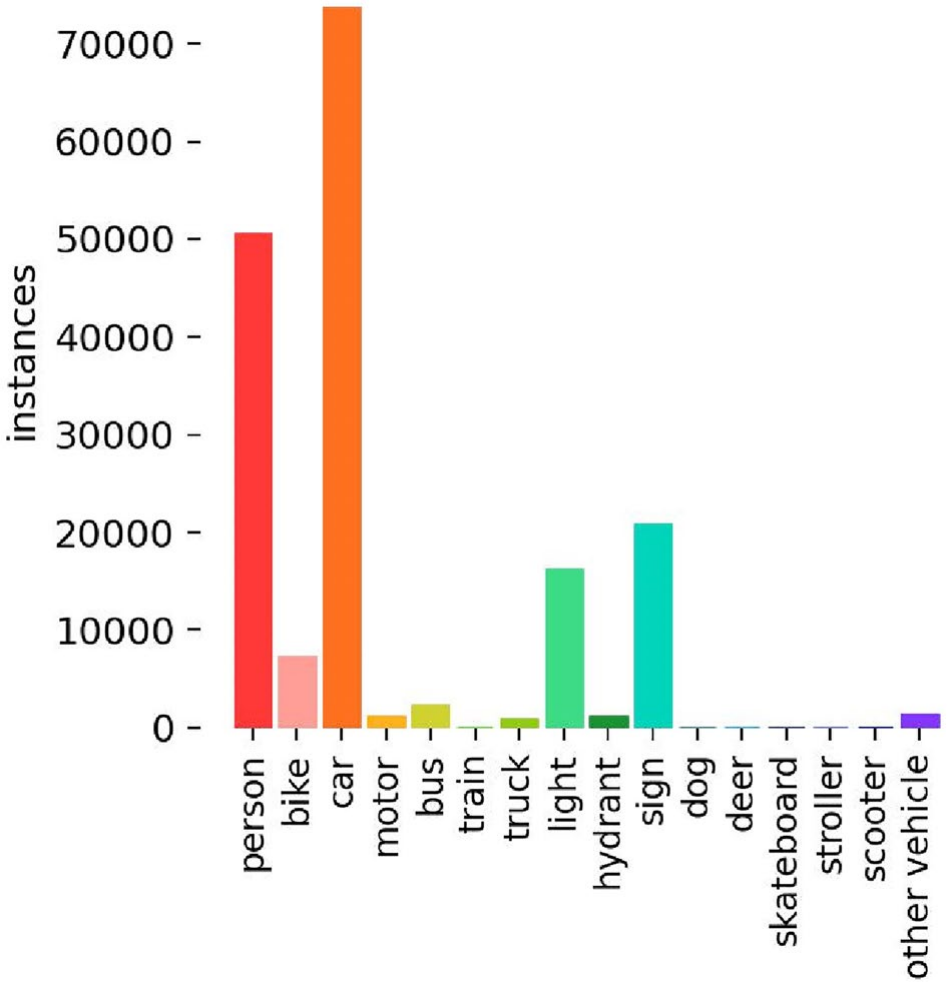
The number of instances of each class in FliR_Adas_v2 dataset.

**Figure 6:**
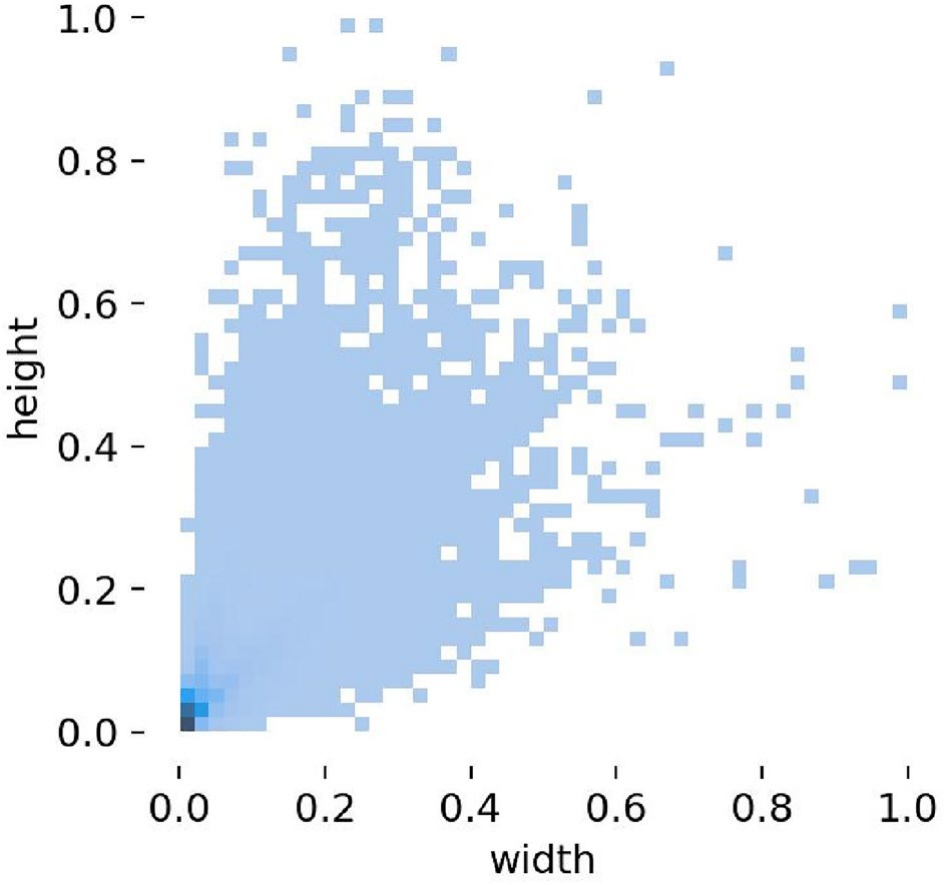
Training set object size of FliR_Adas_v2 dataset.

Infrared image dataset: we use the FliR_Adas_v2 public dataset. This dataset is captured by a camera on a car, with the scene of the car driving on the street. The training set consists of 10,742 images, of which 10,000 are from short video clips, and 724 are from a 140 s video segment, including 16 categories: person, bike, car, motor, bus, train, truck, light, hydraulic, sign, dog, deer, skateboard, roller, scooter, and other vehicles and the number of instances of each class as shown in Fig. 5. In categories with more instances, there are 50,130 people, 7982 bicycles, 73,650 cars, 15,900 lights, and 22,060 signs. The test set consists of 1,144 images, including 11 categories: person, bike, car, motor, bus, truck, light, hydrant, sign, stroller, other vehicle. According to the distribution of the aspect ratios of objects with the same center point in the FliR_Adas_v2 training dataset(as shown in Fig. 6), it can be observed that the aspect ratios of objects are mainly distributed within 0.4 of the input image size. Additionally, there is a dense distribution of extremely small objects within 0.05 of the image size. The challenges in object detection in the FliR_Adas_v2 dataset are as follows:

1. low contrast between the detected object and background;
2. a large number of objects in a single image;
3. small objects with object size less than 10% of the image size;
4. image blurring caused by vehicles during motion.

**Figure 7:**
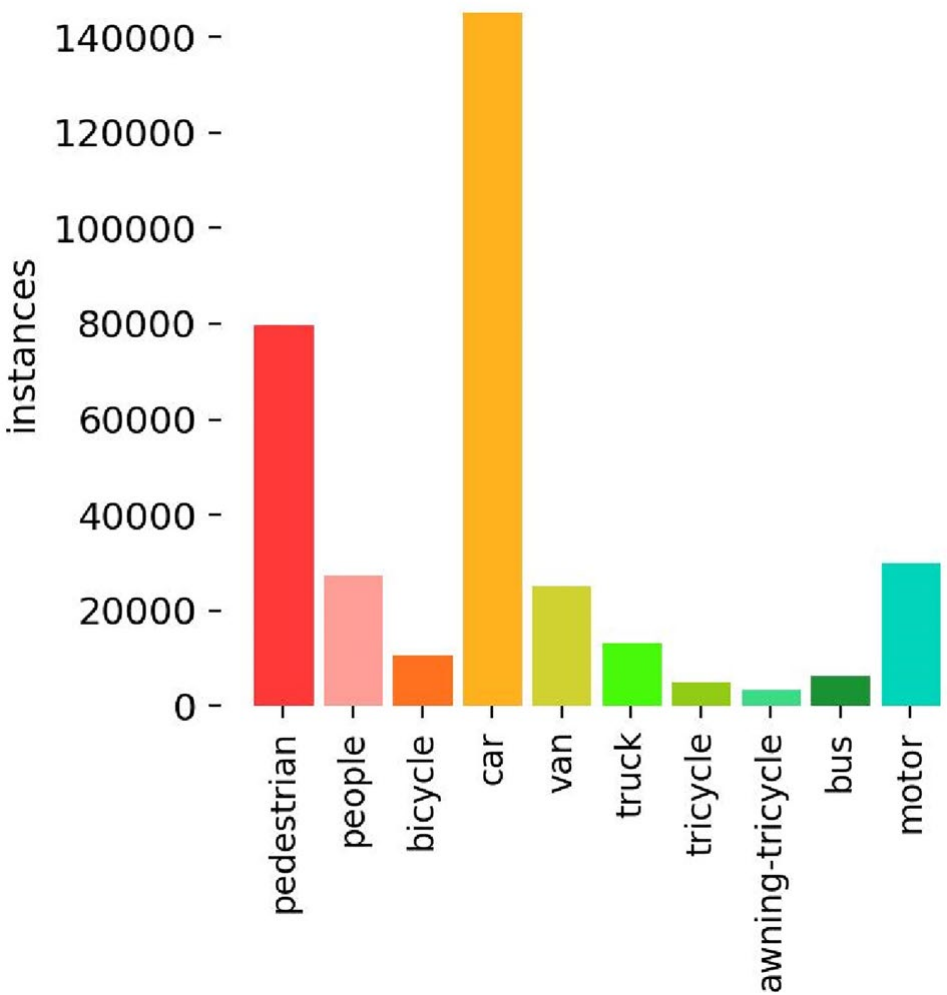
The number of instances of each class in VisDrone2019 dataset.

**Figure 8:**
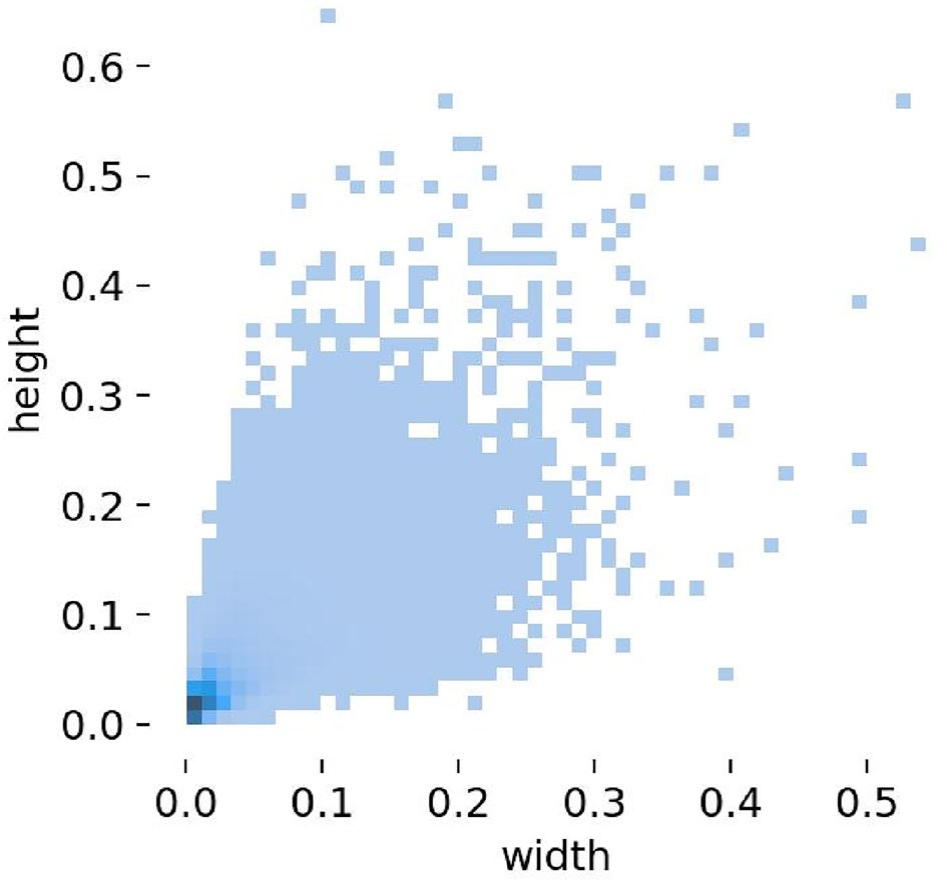
Training set object size of VisDrone2019 dataset.

Visible light image dataset: This paper uses the VisDrone2019 public dataset^45^, which consists of 6,471 training images, 548 validation images, and 3,190 test images (including 1580 images from VisDrone2019-DET-test-challen and 1610 images from VisDrone2019-DET-test-dev). The dataset is captured by various drone cameras and covers a wide range, including location (from 14 different cities across thousands of kilometers in China), environment (urban and rural), objects (pedestrians, vehicles, bicycles, etc.), and density (sparse and crowded scenes). And it contains 10 classes of detection targets, namely pedestrian, people, bicycle, car, van, truck, tricycle, awning-tricycle, bus, and motor, and the number of instances of each class as shown in Fig. 7. In categories with more instances, there are 142,300 cars, 69,800 pedestrians, 24,320 people, 23,390 vans, and 36,600 motors. According to the distribution of the aspect ratios of objects with the same center point in the VisDrone2019 training dataset(as shown in Fig. 8), it can be observed that the aspect ratios of objects are mainly distributed within 0.3 of the input image size. Additionally, there is a dense distribution of extremely small objects within 0.05 of the image size. The challenges in object detection in the VisDrone2019 dataset are as follows:

1. random changes in object size and shape;
2. small objects with object size less than 10% of the image size;
3. the object is often obstructed by other objects, resulting in only partial object information being visible;
4. images typically have large scales and high resolutions, requiring higher computational power;
5. contains various types of objects and complex background environments.

### Implementation details

This experiments were conducted on an Ubuntu 18.04 system with an Intel(R) Xeon(R) Gold 6320R CPU @2.10 GHz,128 GB RAM, NVIDIA GeForce RTX 3090 GPU, and torch version 2.0.0. Parameter settings during training: the image resolution for training models is $640\times 640$. We use mosaic data augmentation and mixup data augmentation; we set lr = 0.01 and training 90 epochs.

### Ablation study

To demonstrate the effectiveness of CDYL, we chose YOLOv8l as the baseline network and added CFC-DC to the backbone network and neck network. The experimental results on the infrared dataset are shown in the Table 1, and the experimental results on the visible light dataset are shown in the Table 2.

**Table 1:** Ablation experiments on FliR_Adas_v2.

Baseline	Mish	CFC-DC	SiLu	Modality	mAP0.5	Flops
✓	✗	✗	✓	IR	53.4	164.9G
✓	✓	✗	✗		53.9	164.9G
✓	✗	✓	✓		56.1	158.8G
(Ours)✓	✓	✓	✗		56.4	158.8G

**Table 2:** Ablation experiments on VisDrone2019.

Baseline	Mish	CFC-DC	SiLu	Modality	mAP0.5	Flops
✓	✗	✗	✓	RGB	41.8	164.9G
✓	✓	✗	✗		42.4	164.9G
✓	✗	✓	✓		42.5	158.8G
(Ours)✓	✓	✓	✗		42.9	158.8G

Through ablation experiments, it was concluded that in infrared image object detection tasks, the baseline network reaches 53.4% in mAP0.5. The activation function of the baseline network is replaced by the Mish activation function, which achieved a 0.5% improvement in mAP0.5. This indicates that the Mish activation function makes the network more sensitive to the detailed information of infrared images. After replacing the core operator with CFC-DC, the network achieved a 2.7% improvement in mAP0.5 compared to the baseline network, indicating that our proposed core operator significantly improved the defects of regular convolution in infrared image object detection tasks; On the basis of the core operator being CFC-DC, adding the Mish activation function reduces network computation while achieving a 56.4% in mAP0.5, which achieved a 3.0% improvement higher than the baseline network. This shows that CDYL has positive performance in infrared target detection tasks.

In the visible light image target detection task, the baseline network reached 41.8% in mAP0.5, and the activation function of the baseline network was replaced by the Mish activation function. The network achieved a 0.6% improvement in mAP0.5 without increasing computational complexity, indicating that the mish function is more sensitive to the information of small targets in the visible light image. After replacing the core operator with CFC-DC, achieved a 0.7% improvement in mAP0.5 compared to the baseline network while reducing network computation, indicating that our proposed core operator has improved perception ability for complex image information compared to regular convolution and has a larger receptive field; Adding a Mish activation function on top of the core operator CFC-DC reduces network computation while achieving a 42.9% in mAP0.5, achieving a 1.1% improvement in mAP0.5 compared to the baseline network. It can be seen that CDYL has positive performance in visible light image object detection tasks.

In order to compare the performance differences between the CFC-DC and convolution with large kernel size, we chose a $7 \times 7$ convolution kernel for the experiment when the parameter and computational complexity of CFC-DC were lower than $7 \times 7$ convolution kernel. The experimental results are shown in Table 3.

**Table 3:** Comparison of detection performance between CFC-DC and $7 \times 7$ convolution kernel.

Baseline	CFC-DC	$7 \times 7$ convolution	Modality	Parameter(M)	Flops(G)	mAP0.5
✓	✗	✗	IR	43.6	164.9	53.4
✓	✓	✗		62.5	158.8	56.4
✓	✗	✓		104.5	260.6	53.9
✓	✗	✗	RGB	43.6	164.9	41.8
✓	✓	✗		62.5	158.8	42.9
✓	✗	✓		104.5	260.6	41.3

Through the experimental results, it was concluded that $7 \times 7$ convolution kernel has increased the number of parameters by 67% and the computational complexity by 64% compared to CFC-DC. In infrared and visible light image detection tasks, CFC-DC achieved 2.5% and 1.6% improvement in mAP0.5 respectively compared to $7 \times 7$ convolution kernel. Although convolution with large kernel size has a larger receptive field, its detection performance is not significantly improved compared to baseline networks and CFC-DC, and its performance is even worse in visible light detection tasks. The improvement of network performance by CFC-DC is not related to the increase in parameter quantity, but rather preserves long-distance dependencies in the network and achieves adaptive spatial aggregation.

The experimental results show that:

1. CDYL has positive performance in target detection tasks in both infrared and visible light images, and has positive generalization ability.
2. CFC-DC can preserve long-range dependencies and achieve spatial adaptive aggregation, reducing computational costs and improving network efficiency without affecting detection accuracy.

### Comparison of detection results of different object detection algorithms on FliR_Adas_v2 and VisDrone 2019

To demonstrate the effectiveness of CDYL in object detection tasks in infrared and visible light images, we conducted a series of comparative experiments. In the infrared image object detection task, because we used YOLOv8l as the baseline network, we first selected some advanced real-time object detection methods for experiments, including YOLOX-l. And, we also selected some object detection methods with slightly lower real-time performance but higher accuracy for experiments, including Faster R-CNN and Mask R-CNN. We used the same training setting as CDYL, using the same learning rate, SGD optimizer, and resolution on the FliR_Adas_v2 dataset. The learning rate is set to 0.01, and the final results are shown in the Table 4.

**Table 4:** Detection results of different methods on FliR_Adas_v2.

Method	Modality	mAP0.5	Latency
Baseline	IR	53.4	12.6 ms
YOLOX-l		48.5	12.7 ms
Faster R-CNN		44.5	21.3 ms
Mask R-CNN		47.0	20.9 ms
CDYL (ours)		56.4	10.6 ms

From the results in the Table 4, it can be seen that our algorithm has positive detection performance on infrared datasets, with a 56.4% in mAP0.5. Compared to the two-stage algorithms Faster R-CNN and Mask R-CNN, our algorithm has achieved 11.9% and 9.4% improvement in mAP0.5, respectively. Compared to the baseline networks YOLOv8l and YOLOX-l, our algorithm has achieved 3.0% and 7.9% improvement in mAP0.5, respectively. From this, it can be seen that our algorithm has positive performance in dealing with infrared image object detection tasks.

We also selected some networks that performed well in visible light image object detection tasks, including YOLOv5l, Faster R-CNN, and CDNet. We used the same training setting as CDYL to train and test on the Visdrone2019 dataset, and the results are shown in the Table 5.

**Table 5:** Detection results of different methods on VisDrone2019.

Method	Modality	mAP0.5	Latency
Baseline	RGB	41.8	13.6 ms
YOLOv5l		37.9	15.1 ms
Faster R-CNN		21.8	24.6 ms
CDNet		34.2	22.9 ms
CDYL (ours)		42.9	11.3 ms

From the results in the Table 5, it can be seen that CDYL performs well in visible light target detection tasks, with a 42.9% in mAP0.5 on the test set, achieving a 21.1% improvement in mAP0.5 compared to Faster R-CNN, and achieving a 1.1% improvement in mAP0.5 compared to the baseline network YOLOv8l.

According to the experimental results in Tables 4 and 5, our algorithm has the lowest latency in infrared and visible light target detection tasks, reaching 10.6 ms and 11.3 ms respectively, and has the highest efficiency while ensuring high accuracy.

To verify the generalization of CFC-DC, we added this module to other object detection networks. We have selected some networks that perform well in infrared and visible light image object detection. The experimental results are shown in the Table 6.

**Table 6:** The generalization of CFC-DC.

Methods	CFC-DC	Modality	mAP0.5
Mask R-CNN	✗	IR	47.0
	✓		48.6
CDNet	✗	RGB	34.2
	✓		35.6

Through the experimental results, we can see that in infrared image target detection, Mask R-CNN applied CFC-DC achieving a 1.6% improvement in mAP0.5, and in visible light image target detection, CDNet applied CFC-DC module achieving a 1.4% improvement in mAP0.5. The experiment shows that CFC-DC performs well in other object detection networks and it has good generalization ability.

Through the above comparative experiments, CDYL has shown positive performance in both infrared and visible light image object detection tasks, and has significantly improved accuracy compared to the current popular object detection networks. CDYL exhibits high efficiency and generalization.

We also compare the parameter size, computation amount, as shown in Table 7. Our algorithm has more parameters than other networks, but its computation amount is lower, which is of great significance for application on edge devices with limited computing power such as drones or cars.

**Table 7:** The parameter size, computation amount of different algorithms.

Method	Parameter(M)	FLOPs(G)
Baseline	43.6	164.9
YOLOX-l	54.2	155.6
YOLOv5l	46.1	107.8
CDYL (ours)	62.5	158.8

**Figure 9:**
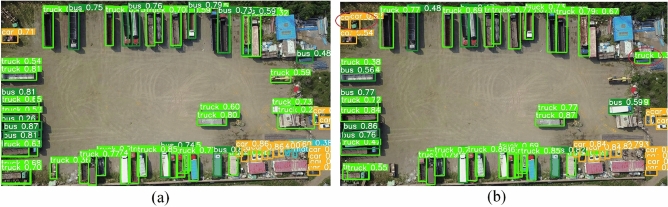
Detection results of YOLOv8l and our proposed algorithm in visible light images. (**a**) Are for our proposed algorithm, (**b**) are for YOLOv8l. Red circles indicate false detection or missed detection.

**Figure 10:**
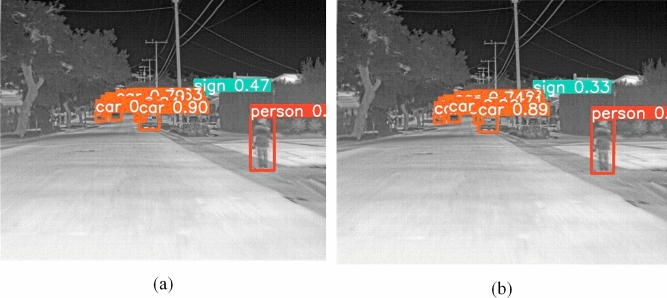
The detection results of our proposed algorithm and baseline network in infrared images. Due to the limitations of two-dimensional display, (**a**) represents the detection results of our proposed algorithm, including 1 person, 12 cars, and 1 sign; (**b**) represents the detection results of baseline network, including 1 person, 10 cars, and 1 sign.

Part of the image detection results are shown in the Figs. 9 and 10. For cars and people with a large sample size, both the baseline network and our algorithm can effectively detect them, and our algorithm has a higher confidence level; For targets that are difficult to detect, the original baseline network may skip over or false detect some targets, but our algorithm can detect them more effectively.

## Conclusion

We propose a new dense small object detection algorithm CDYL for infrared and visible light images, which can be well applied in object detection tasks. Both infrared and visible light images have their own advantages. By fusing the two types of images, rich details of visible light images can be obtained, and the anti-interference ability of infrared images can be obtained, thus obtaining better applications.

We have proposed a new core operator CFC-DC based on the requirements of the task, and added the most suitable Mish activation function to this core operator. A large number of infrared and visible light image object detection experiments have verified that CDYL has positive performance. Compared to the baseline network, our proposed algorithm has achieved 3.0% improvement in mAP0.5 on infrared images and 1.1% improvement in maAP_0.5_ on visible light images. Its low computing cost also ensures its application on edge devices with limited computing power.

## Data Availability

The datasets generated and/or analysed during the current study are not publicly available due to the confidentiality involved in this study but are available from the corresponding author on reasonable request.
